# Accuracy of Samsung Gear S Smartwatch for Activity Recognition: Validation Study

**DOI:** 10.2196/11270

**Published:** 2019-02-06

**Authors:** Anis Davoudi, Amal Asiri Wanigatunga, Matin Kheirkhahan, Duane Benjamin Corbett, Tonatiuh Mendoza, Manoj Battula, Sanjay Ranka, Roger Benton Fillingim, Todd Matthew Manini, Parisa Rashidi

**Affiliations:** 1 Department of Biomedical Engineering University of Florida Gainesville, FL United States; 2 Department of Epidemiology University of Florida Gainesville, FL United States; 3 Department of Computer and Information Science and Engineering University of Florida Gainesville, FL United States; 4 Department of Aging and Geriatric Research University of Florida Gainesville, FL United States; 5 Department of Health Outcomes and Biomedical Informatics University of Florida Gainesville, FL United States; 6 Department of Community Dentistry and Behavioral Science University of Florida Gainesville, FL United States

**Keywords:** actigraphy, activity recognition, machine learning, metabolic equivalent, physical activity

## Abstract

**Background:**

Wearable accelerometers have greatly improved measurement of physical activity, and the increasing popularity of smartwatches with inherent acceleration data collection suggest their potential use in the physical activity research domain; however, their use needs to be validated.

**Objective:**

This study aimed to assess the validity of accelerometer data collected from a Samsung Gear S smartwatch (SGS) compared with an ActiGraph GT3X+ (GT3X+) activity monitor. The study aims were to (1) assess SGS validity using a mechanical shaker; (2) assess SGS validity using a treadmill running test; and (3) compare individual activity recognition, location of major body movement detection, activity intensity detection, locomotion recognition, and metabolic equivalent scores (METs) estimation between the SGS and GT3X+.

**Methods:**

To validate and compare the SGS accelerometer data with GT3X+ data, we collected data simultaneously from both devices during highly controlled, mechanically simulated, and less-controlled natural wear conditions. First, SGS and GT3X+ data were simultaneously collected from a mechanical shaker and an individual ambulating on a treadmill. Pearson correlation was calculated for mechanical shaker and treadmill experiments. Finally, SGS and GT3X+ data were simultaneously collected during 15 common daily activities performed by 40 participants (n=12 males, mean age 55.15 [SD 17.8] years). A total of 15 frequency- and time-domain features were extracted from SGS and GT3X+ data. We used these features for training machine learning models on 6 tasks: (1) individual activity recognition, (2) activity intensity detection, (3) locomotion recognition, (4) sedentary activity detection, (5) major body movement location detection, and (6) METs estimation. The classification models included random forest, support vector machines, neural networks, and decision trees. The results were compared between devices. We evaluated the effect of different feature extraction window lengths on model accuracy as defined by the percentage of correct classifications. In addition to these classification tasks, we also used the extracted features for METs estimation.

**Results:**

The results were compared between devices. Accelerometer data from SGS were highly correlated with the accelerometer data from GT3X+ for all 3 axes, with a correlation ≥.89 for both the shaker test and treadmill test and ≥.70 for all daily activities, except for computer work. Our results for the classification of activity intensity levels, locomotion, sedentary, major body movement location, and individual activity recognition showed overall accuracies of 0.87, 1.00, 0.98, 0.85, and 0.64, respectively. The results were not significantly different between the SGS and GT3X+. Random forest model was the best model for METs estimation (root mean squared error of .71 and r-squared value of .50).

**Conclusions:**

Our results suggest that a commercial brand smartwatch can be used in lieu of validated research grade activity monitors for individual activity recognition, major body movement location detection, activity intensity detection, and locomotion detection tasks.

## Introduction

Wearable accelerometers have greatly improved the objective measurement of physical activity over the past 20 years [1]. They have enabled the detection and tracking of activity intensity and patterns (eg, bouts) in the population and during intervention studies [2-5]. Most research uses accelerometers that have been specifically designed to record accelerations to quantify the amount of time spent in performing activities of different intensities, which is important for understanding the health benefits of physical activity. However, there has been a rapid growth of smartwatches that collect accelerations for both usability purposes (eg, screen orientation) and for tracking activity patterns. In fact, the smartwatch market is expected to grow at an annual rate of 18% through 2021 [6,7], and this allows an unprecedented opportunity to evaluate activity patterns without using a dedicated research device.

Compared with dedicated devices, smartwatches contain some conventional sensors such as heart rate sensors, Global Positioning Systems, and ultraviolet exposure, but also more novel utilities such as a speaker, microphone, and Global System for Mobile Communications data plan for communications. These additional sensors and utilities open new opportunities coupling companion measures along with activity patterns that can be continuously uploaded through wireless networks. Their multitasking platform and increasing popularity make smartwatches an ideal tool for researchers to monitor physical activity in real time without requiring users to wear any additional, dedicated device. For smartwatches to be acceptable in research, the accelerometer needs to be validated and data need to be compared with existing research-grade monitors.

In this study, we validate the triaxial accelerometer in the Samsung Gear S smartwatch (SGS) that currently makes up 16% of the smartwatch market [8]. This is necessary to ensure data from this device is acceptable for objectively measuring time spent in performing activities of different intensities and for recognizing physical activity type. First, the SGS underwent validation on a mechanical shaker table, and raw data were compared against an Actigraph GT9X. Next, a series of experiments were performed on a treadmill and during common daily activities. High-resolution raw accelerometer data were used to extract frequency- and time-domain features that are used to train and test classification models for activity recognition tasks. We compared the accuracies of the SGS and Actigraph GT3X+ (GT3X+) for assessing (1) activity intensity level, (2) locomotion versus nonlocomotion, (3) location of major body movement during the activity, (4) sedentary versus nonsedentary, and (5) individual activity recognition classification. Moreover, a portable metabolic unit was used to record metabolic equivalent scores (METs) to estimate activity intensity and used to further validate the SGS against the Actigraph. We hypothesized that the SGS would test valid and be accurate at assessing activity intensity and recognize activity type as compared with GT3X+.

## Methods

### Data Collection

We collected data in 3 different experiment setups: (1) a shaker table, (2) treadmill walking, and (3) 15 daily activities. In all experiments, we collected data simultaneously from both SGS [9] and GT3X+. Device characteristics are compared and described in Table 1 [10,11]. All study procedures were approved by the University of Florida Institutional Review Board. All participants provided written informed consent before participation in the study. First, we collected data using a mechanical 1-dimensional shaker table by applying acceleration to both devices for 3 min for 7 different speeds (0.5 Hz, 1 Hz, 1.5 Hz, 2 Hz, 2.5 Hz, 3 Hz, and 3.5 Hz), repeated for each axis. SGS monitor was positioned on top of the GT3X to ascertain that they experience the same accelerations. Then, we collected data during 6 speeds of treadmill walking, where 1 participant wore both devices on the right wrist and ambulated at 6 different speeds (1, 2, 3, 4, 5, and 6 mph), 3 min each at each speed. Data were collected at a frequency of 10 Hz for the SGS and 100 Hz for GT3X+ for both the shaker table and treadmill test.

In the third experiment, participants wore both devices on their right wrist while performing several daily activities, as listed in Table 2 [12]. Expiratory gas was collected during each activity using a portable, chest-worn, indirect calorimeter (Cosmed K4b2; COSMED USA). Energy expenditure was estimated using oxygen uptake (VO_2_=milliliter min^−1^kg^−1^) at a steady state, generally beginning at 3 min after starting the activity. Oxygen consumption was subsequently converted to METs, a value that is often used to gauge the intensity of an activity relative to a reference resting value (ie, 3.0 METs=3 times the equivalent of resting oxygen consumption). METs were calculated as the oxygen consumption per minute relative to body mass (ml/min/kg) divided by a resting value of 3.5 ml/min/kg [12,13]. We linked the resulting MET value for each task to the average of the extracted features for the task.

**Table 1:** Technical specifications of ActiGraph GT3X+ and Samsung Gear S smartwatch.

Characteristics	ActiGraph GT3X+	Samsung Gear S smartwatch
Dimensions	4.6 cm x 3.3 cm x 1.5 cm	5.8 cm x 4.0 cm x 1.2 cm
Weight	19 gm	67 gm
Sampling rate	100 Hz	100 Hz
Dynamic range	±8G	±2G
Memory	4 GB	4 GB

**Table 2:** Characteristics of each activity. Accelerometer data were collected from 40 participants.

Activity	Major body movement location	Intensity	Locomotion	Duration (min)	Participants (n)
Computer work	Upper	Sedentary	No	8	11
Ironing	Upper	Light	No	8	12
Yoga	Total	Light	No	8	8
Shopping	Total	Light	No	8	13
Laundry	Upper	Light	No	8	9
Washing windows	Upper	Moderate	No	8	15
Home maintenance	Upper	Moderate	No	8	14
Replacing bed sheet	Upper	Moderate	No	8	13
Mopping	Upper	Moderate	No	8	13
Trash removal	Total	Moderate	No	8	13
Heavy lifting	Total	Moderate	No	8	12
Leisure walk	Lower	Moderate	Yes	8	9
Rapid walk	Lower	Moderate	Yes	8	9
Walk at RPE^a^ 1	Lower	Moderate	Yes	5	14
Walk at RPE 5	Lower	Moderate	Yes	5	13

^a^RPE: ratings of perceived exertion.

**Inclusion criteria for the study.**

1. Community-dwelling adults aged 20 years or older
2. Willingness to undergo all testing procedures
3. Weight stable for at least 3 months (±5 lbs)
4. English speaking

A total of 40 community-dwelling adults aged between 20 and 85 years (n=12 males, mean age 55.2 [SD 17.8] years, mean body mass index 26.8 [SD 6.2] kg/m^2^), participated in this study. These 40 participants belonged to a subset of a larger study whose primary aim was to assess the age effect on energy expenditure during activities common to daily life in the United States [12]. The inclusion criteria are provided in Textbox 1. For a complete list of exclusion criteria refer to [12].

The activities in our study are common daily activities. They included ironing, yoga, shopping, laundry washing, computer work, washing windows, home maintenance, replacing bedsheet, mopping, trash removal, heavy lifting, and walking at 2 different ratings of perceived exertion (RPE), as well as leisure and rapid walking [12]. Activity instructions and the setup of the experiments are described in more detail in the study by Corbett et al [12]. Participants performed activities based on instructions, in a laboratory setting, each for 8 min, except for the walking activities performed at different RPE [14,15]. Activities were designed to be repeated to achieve an accumulated duration of 8 min.

### Analysis

**Figure 1:**
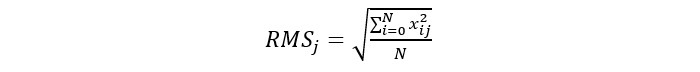
Root mean square (RMS) shows the arithmetic mean of the squares of the accelerometer values.

To validate data from the SGS against data from the GT3X+, we calculated the root mean square (RMS) value of the accelerometer data over 1 second, for all 3 axes, for each device. RMS is calculated using Equation 1 (Figure 1).

Here, *N* is the number of data points and *x*_*ij*_ refers to a single data point *i* from axis *j*. In this step, we calculated the RMS of 1-second windows of the data in each axis. Then, we computed the correlation between the data from the 2 devices along each axis. This process was performed for the shaker table test data, treadmill test data, and daily activity data. We used the middle 2 min of 3-min shaker table data, the middle 2 min of 3-min treadmill data, and the middle 5 min of the 8-min daily activity tests. The purpose of this selection was to exclude the boundary start and end segments of activities.

**Figure 2:**
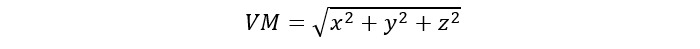
Vector magnitude (VM) here is defined as Euclidean norm of the vector from the origin to the point shown by x, y, and z.

We extracted 15 time- and frequency-domain features found in the current literature (Table 3) [16,17]. Here, vector magnitude is defined as in Equation 2 (Figure 2), where x, y, and z are accelerations in the Cartesian coordinate system. In addition to the features previously suggested [16], our feature set also included (1) kurtosis, which is a descriptor of the shape of the distribution of the acceleration data in each window; (2) skewness, which measures the asymmetry of the distribution of acceleration data in the window; and (3) entropy, which helps in discriminating between activities with similar power spectral density but different movement patterns [17].

The length of the window of data used for statistical feature calculation is also important [18,19]. Previous individual activity recognition studies have used different window lengths, ranging from 0.1 seconds to 128 seconds [16,20-24]. In this study, we do not use window lengths smaller than 1 second because we are using frequency-domain features. We calculated the statistical features for 6 commonly used window lengths (1-, 2-, 4-, 8-, 15-, and 16-second lengths), to choose the best window length for our classification tasks. These window lengths were chosen in accordance with previous literature from the above list, limited to between windows smaller than 16 seconds. We did not go above the 16-second length as it would drastically reduce the number of sample points and will potentially include different actions in a single window of an activity. We used the overall accuracy metric to select the best model. The features calculated for the chosen window length are used in the prediction models.

Classification tasks included (1) detection of the location of major body movement, (2) detection of activity intensity level, (3) detection of locomotion, (4) detection of sedentary activity, and (5) individual activity recognition for the 15 daily activities.

**Table 3:** Description of the features extracted from the raw data.

Feature	Description
Mean of vector magnitude (MVM)	Sample MVM in the window
Standard deviation of vector magnitude (SDVM)	SDVM in the window
Percentage of the power of the vector magnitude (VM) that is in the range of 0.6-2.5 Hz	Sum of moduli corresponding to frequency in this range/sum of moduli of all frequencies
Dominant frequency (DF) of VM	Frequency corresponding to the largest modulus
Fraction of power in VM at DF	Modulus of the dominant frequency divided by sum of moduli at each frequency
Mean angle of acceleration relative to vertical on the device	Sample mean of the angle between x-axis and VM in the window
SD of the angle of acceleration relative to vertical on the device	Sample SD of the angles in the window
Covariance	Covariance of the VM in the window
Skewness	Skewness of the VM in the window
Kurtosis	Kurtosis of the VM in the window
Entropy	Entropy of the VM in the window
Coefficient of variation	SD of VM in the window divided by the mean, multiplied by 100
Corr(x,y)	Correlation between x-axis and y-axis
Corr(y,z)	Correlation between y-axis and z-axis
Corr(x,z)	Correlation between x-axis and z-axis

**Figure 3:**
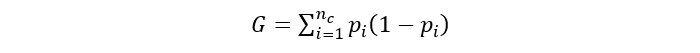
Gini impurity measures the likelihood of incorrect classification of a randomly selected instance of a set, if it were randomly classified according to the distribution of class labels from the set.

The activities in the study include both simple and complex activities [25]. Simple activities, which include walking and computer work in our dataset, consist of repeating a single action to complete the activity. Complex activities, which include ironing, yoga, shopping, laundry washing, washing windows, home maintenance, replacing bedsheet, mopping, trash removal, and heavy lifting, are composed of several simple actions. For example, trash removal activity consists of sorting the trash, finding smaller trash cans, picking up the trash bags, and taking all the trash to a predetermined location. The heterogeneity of complex activities can significantly degrade the performance of an individual activity recognition classifier, as opposed to recognition of simple individual activities. In this study, we report the overall accuracy for simple activities, complex activities, and all activities as well as the balanced accuracy of each activity. Balanced accuracy is calculated as the arithmetic mean of sensitivity and specificity.

For the classification tasks, we used decision tree, random forest, support vector machines, and neural networks models. These models can flexibly represent various relationships between the features and the outcome and have been previously used for activity type classification tasks [16].

We report the ranking of the features’ importance in the random forest model. The random forest model calculated the importance of the features using the decrease in the Gini index. The Gini impurity index is calculated using Equation 3 (Figure 3). Every time a split is made on a variable, the Gini impurity index for the 2 descendent nodes is less than the Gini impurity index of the parent node. The decrease in the Gini index for each variable is calculated by adding up the Gini decreases for the variables over all trees in the forest.

Here, *n*_*c*_ is the number of classes in the outcome and *p*_*i*_ is the ratio of the class *i*. For the MET estimation regression task, the feature importance values are calculated based on the total decrease in node impurities (difference between the residual sum of squares before and after splitting on the variable), averaged over all trees in the random forest model.

For both classification and regression tasks, we used nested cross-validation for evaluating our models. In each fold, we divided the data into 20% test data and 80% development data. The development data was further divided into 20% validation data and 80% training data. Validation data are used for tuning the parameters of the models and removing collinear features. Data partitioning was based on each feature extracted from the window length, rather than by partitioning based on participants, as individual participants did not have all the tasks, and separating based on participants might lead to the absence of some of the classes in some classification tasks, for example, individual activity recognition classification task. All statistical analyses were performed in R (version 3.1.3) [26].

## Results

**Figure 4:**
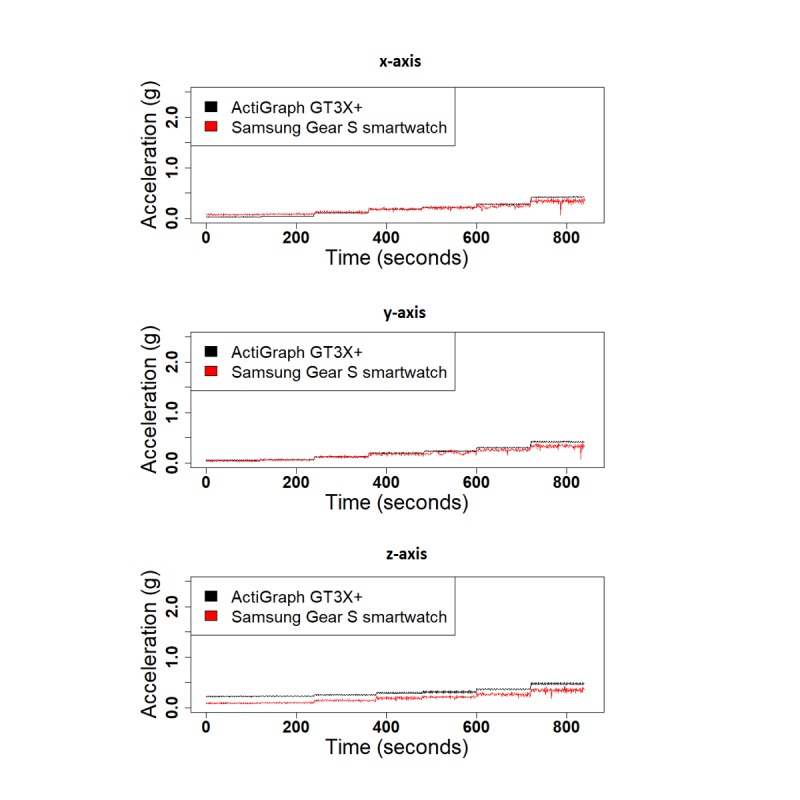
The 1-second root mean square of the acceleration data from Samsung Gear S smartwatch and ActiGraph GT3X+ for each axis for the shaker table.

**Figure 5:**
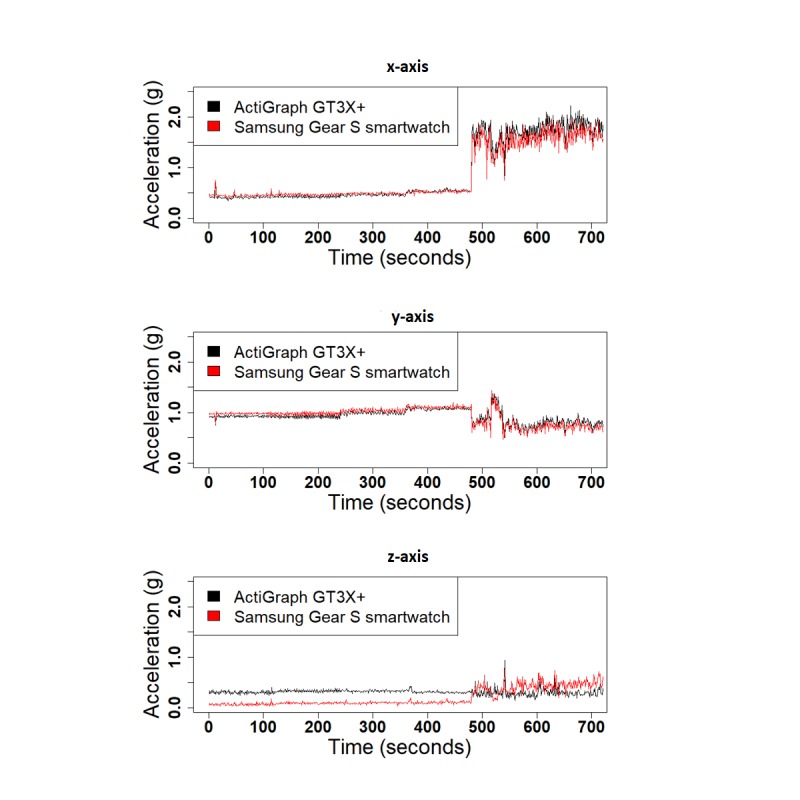
The 1-second root mean square of the acceleration data from Samsung Gear S smartwatch and ActiGraph GT3X+ for each axis for the treadmill test.

**Figure 6:**
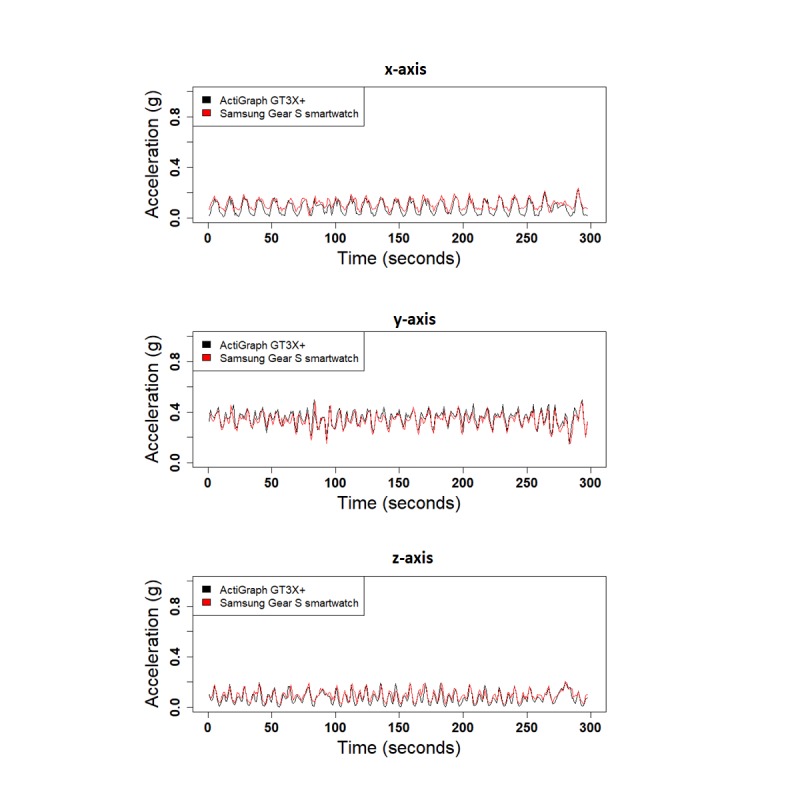
Acceleration data from leisure walking (simple activity) for all 3 axes.

**Figure 7:**
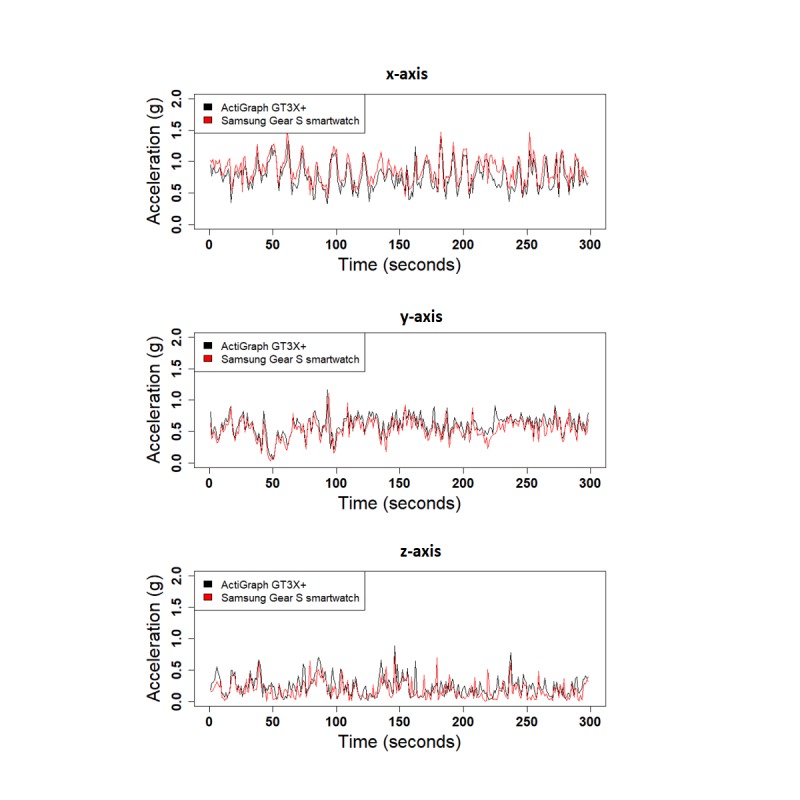
Acceleration data from mopping (complex activity) for all 3 axes.

We performed 3 different experiments to evaluate the validity of SGS in comparison with the GT3X+: (1) shaker table, (2) treadmill, and (3) daily activities. Figure 4 shows the 1-second RMS of the raw acceleration data collected from both devices along the 3 x-, y-, and z-axes for the shaker table test and treadmill test. Correlation values for the 1-second RMS for all 3 axes were high for both the shaker table test and treadmill test (x-corr=.97, y-corr=.97, and z-corr=.95 for the shaker table and x-corr=.98, y-corr=.89, and z-corr=.93 for the treadmill test). Acceleration data from the 3 axes are highly correlated, except for small shifts in amplitude (Figure 5). Correlation values between SGS data and GT3X+ for 3 axes are high in daily activities as well (x-corr>.70, y-corr>.70, and z-corr>.71, except for computer work; Table 4). Figure 6 shows the similarity in acceleration measurement for walking activity as an example of simple activities, and Figure 7 shows the similarity in acceleration measurement for mopping as an example of complex activities, along the 3 x-, y-, and z-axes. Figure 6 shows the repeated single actions for a simple activity, and Figure 7 shows the varying actions occurring during a complex activity.

To test the effect of window length, we repeated our classification tasks with features extracted using varying window length (1, 2, 4, 8, 15, and 16 seconds). We initially observed that random forest has the highest overall accuracy. We evaluated the effect of window length used for extracting the features on the performance of the random forest model. Models trained on the features extracted based on larger window length had better performance (Table 5) [18]. We used features extracted from 16-seconds windows for the remainder of the paper.

**Figure 8:**
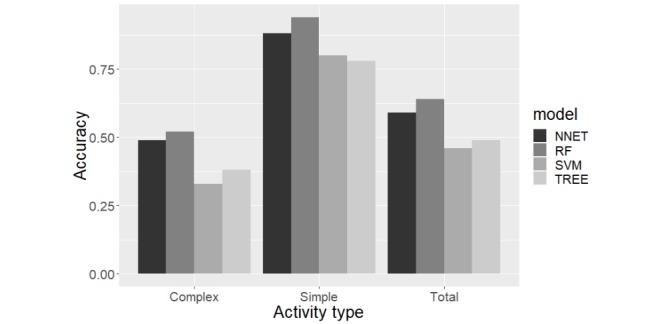
Comparison of the performance of different classifiers for activity recognition task, in terms of overall accuracy for simple tasks, complex tasks, and total set of tasks. TREE: decision tree model, NNET: neural network model, RF: random forest model, and SVM: support vector machines model.

**Figure 9:**
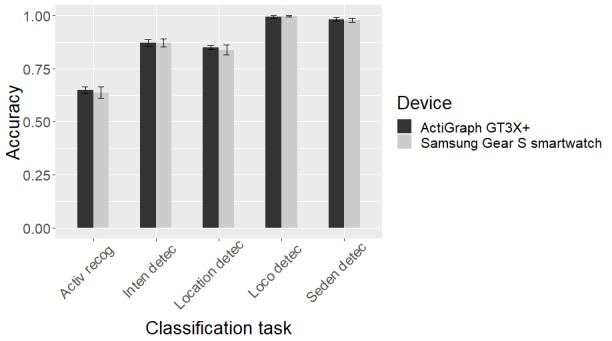
Accuracies of the 5-fold cross-validation classification tasks performed on Samsung Gear S smartwatch data and ActiGraph GT3X+ data. We used the best model and best window length (random forest model and 16-second window length feature extraction). Activ recog: Activity recognition; Inten detec: Activity intensity level detection; Location detec: Major body movement location detection; Loco detec: Locomotion detection; Seden detec: Sedentary activity detection.

**Figure 10:**
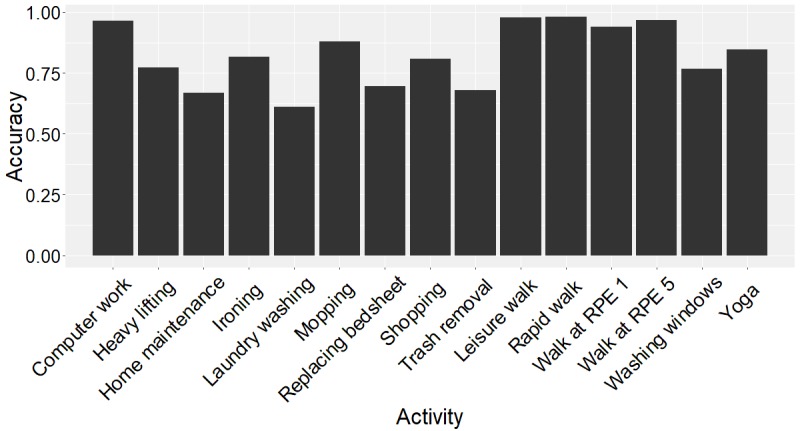
Balanced accuracy for each activity using the random forest model and 16-seconds window for extraction of features. RPE: ratings of perceived exertion.

Figure 8 shows the performance of our different models in terms of the overall accuracy, simple individual activity recognition accuracy, and complex individual activity recognition accuracy. Random forest has the best performance across all 3 activity recognition tasks. Although the random forest model is technically a collection of decision trees, it is designed to correct the overfitting of decision trees. The random forest model generally works better than support vector machines in multi-class classification problems, but the difference is much smaller for binary classification tasks. Figure 9 shows how SGS and GT3X+ perform in our classification tasks. Random forest model’s performance in terms of balanced accuracy of each activity shows that the devices can detect the simple activities—computer work and walking activities—better (Figure 10). Multimedia Appendix 1 gives the normalized confusion matrix of the detected labels versus actual labels of the activities as classified by the random forest model.

**Table 4:** Correlations of 1-second root mean square for all 3 axes of the Samsung Gear S smartwatch and ActiGraph GT3X+ by activity. All correlation values were statistically significant.

Task: correlation		x-axis	y-axis	z-axis
**Simple activities**				
	Computer work	.83	.55	.63
	Leisure walk	.88	.86	.85
	Rapid walk	.78	.70	.76
	Walk at RPE^a^ 1	.79	.92	.87
	Walk at RPE 5	.70	.75	.71
**Complex activities**				
	Shopping	.89	.84	.86
	Mopping	.94	.89	.75
	Home maintenance	.91	.84	.81
	Washing windows	.90	.80	.82
	Heavy lifting	.94	.90	.71
	Ironing	.83	.81	.78
	Replacing bedsheet	.87	.88	.78
	Yoga	.93	.88	.90
	Trash removal	.92	.92	.84
	Laundry washing	.89	.85	.79

^a^RPE: ratings of perceived exertion.

**Table 5:** Effect of window length for feature extraction on micro-averaged accuracy using random forest model for individual activity recognition, locomotion detection, sedentary activity detection, activity intensity level classification, and major body movement location detection. The best performance for each classification task is presented in italics.

Classification task: window length	Classification accuracy (seconds)					
	1	2	4	8	15	16
Individual activity recognition	.45	.47	.56	.60	.64	.64
Locomotion	1	1	1	1	1	1
Sedentary	.96	.97	.97	.98	.98	.98
Intensity	.80	.81	.82	.84	.87	.87
Major body movement location	.78	.80	.81	.83	.85	.85

**Figure 11:**
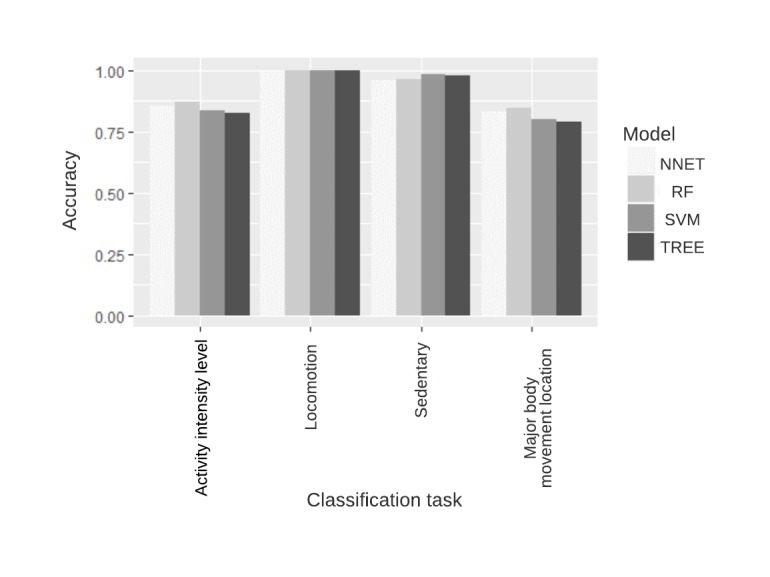
Performance of 4 classifier models in activity type classification tasks in terms of accuracy. NNET: neural networks model; RF: random forest model; SVM: support vector machines model, TREE: decision trees model.

**Figure 12:**
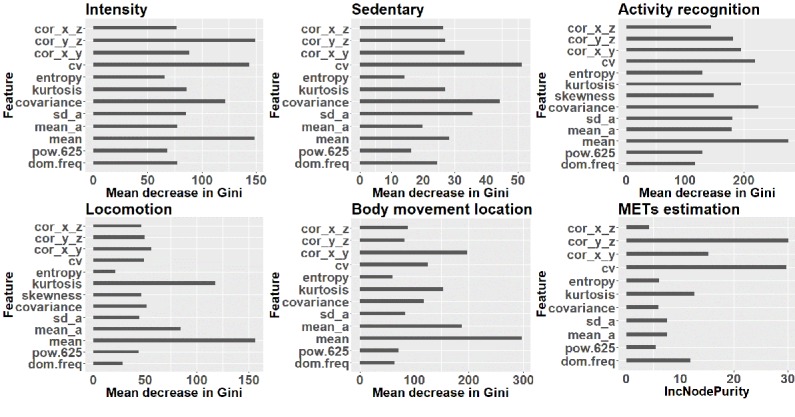
Importance of features in terms of the mean decrease in the Gini index in each classification task and in terms of the increase in node purity for the metabolic equivalent scores estimation regression task, as reported by the random forest model and 16-second window length for feature extraction (note the different range for the x-axis). METs: metabolic equivalent scores; IncNodePurity: Increase in node purity; cor_x_z: correlation between x-axis and z-axis; cor_y_z: correlation between y-axis and z-axis; cor_x_y: correlation between x-axis and y-axis; cv: covariance of the vector magnitude; sd_a: SD of angle; mean_a: mean of angle; pow.625: percentage of the power of the vector magnitude that is in the range of 0.6-2.5 Hz; dom.freq: dominant frequency of vector magnitude.

In the next step, we used our classifiers (decision trees, random forest, support vector machines, and neural networks) on the extracted features to classify the activities based on their intensity, sedentary status, locomotion status, and location of major body movement. The overall accuracies of the models are compared for each classification task in Figure 11. The confusion matrices of the model with the highest accuracy-random forest model for each task are given in Tables 6 to 9. Random forest has the best performance in all tasks based on overall accuracy. Figure 12 shows the ranking of features used in the random forest model for all classification and regression tasks.

**Table 6:** Normalized confusion matrix showing the percentage of correctly classified instances for the random forest model used for activity type classification tasks based on intensity level. Each column shows the actual activity and rows represent the predicted labels.

Activity	Sedentary	Light	Moderate
Sedentary	0.87	0.02	0
Light	0.12	0.65	0.04
Moderate	0.01	0.33	0.95

**Table 7:** Normalized confusion matrix showing the percentage of correctly classified instances for the random forest model used for activity type classification tasks based on locomotion. Each column shows the actual activity and rows represent the predicted labels.

Activity	Locomotion	Nonlocomotion
Locomotion	0.98	0
Nonlocomotion	0.02	1

**Table 8:** Normalized confusion matrix showing percentage of correctly classified instances for the random forest model used for activity type classification tasks based on major body movement location. Each column shows the actual activity and rows represent the predicted labels.

Activity	Lower	Total	Upper
Lower	1	0	0
Total	0	0.61	0.11
Upper	0	0.39	0.89

**Table 9:** Normalized confusion matrix showing percentage of correctly classified instances for the random forest model used for activity type classification tasks based on sedentary. Each column shows the actual activity and rows represent the predicted labels.

Activity	Sedentary	Nonsedentary
Sedentary	0.78	0.01
Nonsedentary	0.22	0.99

Using random forest, support vector machines, neural network, and decision trees models for the regression task of METs estimation shows that the random forest model also has the best performance for the METs estimation. Random forest, decision tree, neural networks, support vector machines models had RMS error values of .71, .77, .99, and .77, respectively. Their r-squared values—which is the coefficient of determination defined as the proportion of the variance in the output that is predictable from the input features—were .50, .40, .01, and .41, respectively.

## Discussion

### Principal Findings

The goal of this study was to validate the SGS accelerometer data against the GT3X+ accelerometer data using several comparisons. First, the accelerometer data from SGS and GT3X+ were compared using a mechanical shaker at different speeds. Second, data were collected and compared from the 2 devices worn by a participant ambulating on a treadmill at different speeds. Third, data collected during activities common in daily life were collected and compared in all 3 axes. We also compared the performance of activity recognition and classification models using accelerometer data recorded using SGS against using accelerometer data recorded using GT3X+.

Accelerometer data from SGS and GT3X+ have high correlations along all 3 axes during shaker tests. However, the correlations were slightly lower when performing daily activities. There are several scenarios where these 2 devices might show different acceleration values. The lower correlation values during the daily activities might partially be caused by nonalignment and movement of the 2 devices during the activities. When the 2 devices are not perfectly aligned, their axes are not pointing in the same direction. This may cause the movement to be dispersed across multiple axes with varying ratios for each device. Another factor that might result in a lower correlation between the 2 devices is the relative slowness of movements during activities such as computer work or yoga, where even small differences are comparatively more significant.

In our study, all 4 models performed well for activity type classification tasks. Random forest had the best performance for all classification tasks. This difference was greater for the individual activity recognition task. Neural networks model’s performance was similar to the random forest model, which were superior to support vector machines, which is more suitable for binary classification, and decision tree model, which tends to overfit [27]. Mean vector magnitude was the most important feature in our tasks, whereas the ranking of other features understandably varied among the different classification tasks. We also used the random forest, decision trees, support vector machines, and neural network models for METs estimation. Random forest had the best performance in this task as well, but other models, except for the neural network, had similar performances.

In this work, we experimented with 1 second, 2 seconds, 4 seconds, 8 seconds, 15 seconds, and 16 seconds nonoverlapping time windows. Larger window lengths resulted in better classification accuracy (Table 5). This may be because of smaller window length capturing shorter duration of actions that are common among different activities. A window length of 16 seconds resulted in high accuracies for activity type classifications such as locomotion versus nonlocomotion. However, it did not lead to high accuracy for individual activity recognition. The lower overall accuracy of classifiers for individual activity recognition can be attributed to the heterogeneous nature of complex activities that are composed of multiple simpler actions under the same label. The fact that the same label here corresponds to different actions deteriorates the performance of the classifier. To remedy this problem for future models, one can separately label every single action in a complex activity.

We observed a slight shift in acceleration amplitude during the mechanical shaker tests between the 2 devices. For our classification purposes, this shift would not affect the models because the relative change in acceleration appeared to be preserved in the SGS. Therefore, it is not expected to impact the classification results in practice. However, a comparison between devices is cautioned until corrections can be made to equate absolute thresholds of accelerations. Overall, our results show that the performances of the SGS and GT3X in various activity recognition tasks and METs estimation are similar and can potentially be used interchangeably between studies. However, the raw data are not interchangeable because of the slight shift explained, and thus, any threshold derived for 1 device needs to be validated for the other device to be compared.

### Limitations

There are limitations that need to be acknowledged. First, inter-device reliability was not tested, given the use of a commercial device. Another limitation is that SGS was evaluated in standard settings (eg, shaker table and laboratory activities) and thus may not be applicable to free-living conditions. In this study, we compared the performances of the machine learning models trained on data collected by the 2 devices in laboratory settings and structured activities. However, free-living activity recognition tasks are more complicated because of factors such as a temporal overlap between the activities, similar action units in several activities, activity fragmentation, and the interpersonal and intrapersonal variation in activities as well as variation in wear locations. Future works can focus on comparing the performance of SGS-based models with GT3X-based models in free-living activity recognition tasks. Such efforts would need to implement methods for activity label determination, such as body-worn camera recordings that allow for later labeling of the activities performed. In addition, the tasks that were evaluated are not representative of all the tasks that an individual may perform. Although the activities constitute a wide range of movements, the results reported in this study are limited to the activities tested.

### Conclusions

In this study, we showed that data collected from a commercial brand smartwatch performed similarly to a research-grade accelerometer to detect a variety of simple and complex activity types. The comparable performance of models relying on SGS and GT3X+ data for activity recognition and energy expenditure estimation verifies the validity of the SGS for research purposes.

## Appendix

Multimedia Appendix 1 Normalized confusion matrix for individual activity recognition task, using the random forest model and 16-second window for extraction of features. Each column shows the actual activity and rows represent the predicted labels. Italicized diagonal elements show the percentage of accurately classified points for each activity.

## Abbreviations

GT3X+: ActiGraph GT3X+
MET: metabolic equivalent score
RMS: root mean square
RPE: ratings of perceived exertion
SGS: Samsung Gear S smartwatch
