# Supplementary material for: Accuracy of Samsung Gear S Smartwatch for Activity Recognition: Validation Study
Source: JMIR Mhealth Uhealth. 2019 Feb 6;7(2):e11270. doi: 10.2196/11270 (PMC6386649; doi:10.2196/11270)
Supplement: Multimedia Appendix 1 [file mhealth_v7i2e11270_app1.docx]

Multimedia Appendix Table 1. Normalized confusion matrix for individual activity recognition task, using the random forest model and 16-second window for extraction of features. Each column shows the actual activity and rows represent the predicted labels. Italicized diagonal elements show the percentage of accurately classified points for each activity.

| Activity | CW | HL | HM | IR | LW | MO | RBS | SH | TR | W11 | W12 | W21 | W22 | WW | YO |
| --- | --- | --- | --- | --- | --- | --- | --- | --- | --- | --- | --- | --- | --- | --- | --- |
|  |  |  |  |  |  |  |  |  |  |  |  |  |  |  |  |
| **Computer Work (CW)** |  |  |  |  |  |  |  |  |  |  |  |  |  |  |  |
|  | *.94* | 0 | 0 | 0 | 0 | 0 | .01 | .06 | 0 | .01 | .01 | 0 | .01 | 0 | .13 |
| **Heavy Lifting (HL)** |  |  |  |  |  |  |  |  |  |  |  |  |  |  |  |
|  | 0 | *.57* | .11 | .01 | .11 | .01 | .06 | .03 | .18 | 0 | 0 | 0 | 0 | .07 | 0 |
| **Home Maintenance (HM)** |  |  |  |  |  |  |  |  |  |  |  |  |  |  |  |
|  | 0 | .09 | *.38* | .04 | .18 | .05 | .13 | .06 | .11 | 0 | 0 | 0 | 0 | .10 | .01 |
| **Ironing (IR)** |  |  |  |  |  |  |  |  |  |  |  |  |  |  |  |
|  | 0 | .01 | .07 | *.65* | .09 | .01 | .02 | .15 | .04 | 0 | 0 | 0 | 0 | .05 | 0 |
| **Laundry Washing (LW)** |  |  |  |  |  |  |  |  |  |  |  |  |  |  |  |
|  | 0 | .01 | .04 | .05 | *.19* | .02 | .04 | .01 | .04 | 0 | 0 | 0 | 0 | .02 | 0 |
| **Mopping (MO)** |  |  |  |  |  |  |  |  |  |  |  |  |  |  |  |
|  | 0 | .02 | .03 | .03 | .10 | *.75* | .03 | .02 | .09 | 0 | 0 | 0 | 0 | .03 | 0 |
| **Replacing Bed Sheet (RBS)** |  |  |  |  |  |  |  |  |  |  |  |  |  |  |  |
|  | 0 | .08 | .13 | .01 | .10 | .04 | *.42* | 0 | .09 | 0 | 0 | 0 | 0 | .04 | .01 |
| **Shopping (SH)** |  |  |  |  |  |  |  |  |  |  |  |  |  |  |  |
|  | .05 | .05 | .07 | .15 | 0 | 0 | 0 | *.61* | .02 | .01 | 0 | .01 | 0 | .07 | .11 |
| **Trash Removal (TR)** |  |  |  |  |  |  |  |  |  |  |  |  |  |  |  |
|  | 0 | .14 | .07 | .02 | .11 | .05 | .11 | .02 | *.37* | 0 | 0 | 0 | 0 | .06 | .01 |
| **Leisure walk (W11)** |  |  |  |  |  |  |  |  |  |  |  |  |  |  |  |
|  | 0 | 0 | 0 | 0 | 0 | 0 | 0 | 0 | 0 | *.95* | 0 | .09 | .01 | 0 | 0 |
| **Rapid walk (W12)** |  |  |  |  |  |  |  |  |  |  |  |  |  |  |  |
|  | 0 | 0 | 0 | 0 | 0 | 0 | 0 | 0 | 0 | .01 | *.96* | 0 | .02 | 0 | 0 |
| **Walk at RPE 1 (W21)** |  |  |  |  |  |  |  |  |  |  |  |  |  |  |  |
|  | 0 | 0 | 0 | 0 | 0 | 0 | 0 | 0 | 0 | .01 | .01 | *.90* | 0 | 0 | 0 |
| **Walk at RPE 5 (W22)** |  |  |  |  |  |  |  |  |  |  |  |  |  |  |  |
|  | 0 | 0 | 0 | 0 | 0 | 0 | 0 | 0 | 0 | .01 | .02 | 0 | *.94* | 0 | 0 |
| **Window washing (WW)** |  |  |  |  |  |  |  |  |  |  |  |  |  |  |  |
|  | 0 | .03 | .09 | .01 | .11 | .05 | .13 | 0 | .04 | 0 | 0 | 0 | 0 | *.56* | 0 |
| **Yoga (YO)** |  |  |  |  |  |  |  |  |  |  |  |  |  |  |  |
|  | .01 | 0 | 0 | 0 | .01 | 0 | .04 | .03 | .02 | .01 | 0 | .01 | 0 | .01 | *.63* |
